# Robotic Intracellular Electrochemical Sensing for Adherent Cells

**DOI:** 10.34133/2022/9763420

**Published:** 2022-09-02

**Authors:** Weikang Hu, Yanmei Ma, Zhen Zhan, Danish Hussain, Chengzhi Hu

**Affiliations:** ^1^Shenzhen Key Laboratory of Biomimetic Robotics and Intelligent Systems, Department of Mechanical and Energy Engineering, Southern University of Science and Technology, Shenzhen, China; ^2^Department of Mechatronics Engineering, National University of Sciences and Technology, Islamabad, Pakistan; ^3^Guangdong Provincial Key Laboratory of Human-Augmentation and Rehabilitation Robotics in Universities, Southern University of Science and Technology, Shenzhen, China

## Abstract

Nanopipette-based observation of intracellular biochemical processes is an important approach to revealing the intrinsic characteristics and heterogeneity of cells for better investigation of disease progression or early disease diagnosis. However, the manual operation needs a skilled operator and faces problems such as low throughput and poor reproducibility. This paper proposes an automated nanopipette-based microoperation system for cell detection, three-dimensional nonovershoot positioning of the nanopipette tip in proximity to the cell of interest, cell approaching and proximity detection between nanopipette tip and cell surface, and cell penetration and detection of the intracellular reactive oxygen species (ROS). A robust focus algorithm based on the number of cell contours was proposed for adherent cells, which have sharp peaks while retaining unimodality. The automated detection of adherent cells was evaluated on human umbilical cord vein endothelial cells (HUVEC) and NIH/3T3 cells, which provided an average of 95.65% true-positive rate (TPR) and 7.59% false-positive rate (FPR) for in-plane cell detection. The three-dimensional nonovershoot tip positioning of the nanopipette was achieved by template matching and evaluated under the interference of cells. Ion current feedback was employed for the proximity detection between the nanopipette tip and cell surface. Finally, cell penetration and electrochemical detection of ROS were demonstrated on human breast cancer cells and zebrafish embryo cells. This work provides a systematic approach for automated intracellular sensing for adherent cells, laying a solid foundation for high-throughput detection, diagnosis, and classification of different forms of biochemical reactions within single cells.

## 1. Introduction

Measurements of intracellular biochemical processes play a significant role in quantitatively understanding the fundamental biological process or cellular heterogeneity in various physiological and pathological conditions [1]. Recent evidence has shown that reactive oxygen species (ROS) play crucial roles in multiple biological processes, including ion transport, signal transduction, cell proliferation, and apoptosis induction [2]. Various intracellular ubiquitous chemical factors such as pH, Ca^2+^, and ROS have been recognized to have a significant association with the initiation and progression of tumors [3, 4]. With the steadfast advance of synthetic biology, monitoring intracellular signals has enabled scientists to elucidate the mechanism of ion homeostasis and signaling, facilitating the use of genetically engineered cells for the development of innovative therapeutics [5].

In the past decades, the increasing availability of sophisticated analytical techniques has expanded the routes for cell exploration [6–9]. Several analytical techniques have emerged to achieve single-cell intracellular sensing, including fluorescence-based spectroscopy/microscopy [10], dark-field scattering microscopy [11], surface-enhanced Raman spectroscopy [12], and nanopore/nanoelectrode sensing [13, 14]. Fluorescence imaging is widely used for subcellular visualization for revealing subcellular processes [15]. However, fluorescent probes are prone to disturb the physiological state of the cells due to cytotoxicity and photobleaching/blinking over long timescales [16]. Meanwhile, the number of live-cell-permeant fluorophores that are available to perform intracellular sensing is still limited. Nanopipette-based electrochemical sensing provides an alternative to fluorescence-based assays by label-free and nondestructive measurement of the intracellular chemical reactivity of the target species [17]. Due to the small size of the tip, the damage to a single cell is minimal. Nanopipette-based electrochemical biosensing has enabled the detection of subcellular processes and intracellular molecules with high sensitivity and spatial resolution because of the limited space at the tip of the nanopipette [18]. Besides, the versatility in the surface chemical modification to the nanopipette has increased the stability, safety, efficiency, and diversity of the detected species [19, 20]. However, the manually targeted intracellular electrochemical sensing for single cells is limited in efficient probing of subcellular processes of the heterogeneous cell populations. In addition, the manual intracellular microoperation is time-consuming and has low repeatability and throughput. Hence, automated and high-throughput sensing systems are of scientific interest to probe intracellular processes with high statistical importance.

Robotic cell manipulation technologies focusing on suspended cells have been developed over the recent years [21–26]. Compared to the suspended cells, cell detection and other automated microoperations are difficult for adherent cells since they are smaller and irregularly shaped [27, 28]. Till now, several groups have achieved semiautomated high-throughput microinjection for adherent cells. By using the semiautomated microinjection systems, both the microinjection pipette and microscope stage are motorized, and microinjection on localized sites is realized by computer mouse clicking [28, 29]. However, manual cell identification (without cell detection capabilities) is still tedious to obtain sufficient data of statistical significance. Pan et al. designed a fully automated high-productivity microinjection system with the capability of cell detection based on fluorescence imaging [30]. However, the fluorescence imaging may lead to cell damage and introduce disturbance to intracellular electrochemical sensing.

Efficient automatic cell targeting and nanopipette tip positioning have been the main features for the development of fully automated microoperation systems. Dewan et al. achieved cell detection based on the *k*-means clustering method [27]. Patino et al. realized nucleus location by a fully convolutional network [31]. The performance of these cell detection algorithms strongly depends on clear cell features in the images. Generally, defocusing will increase the grayscale difference between foreground (cells) and background by reducing the high-frequency components of the image, thus simplifying the cell detection [32]. However, the defocused plane determined by experience cannot ensure the maximum difference between foreground and background, leading to a poor cell detection rate. On the other hand, automatically locating the tip of the nanopipette is critical to avoid any damage to the tip. Youoku et al. [33] and Liu et al. [34] utilized curve fitting algorithm and quadtree recursive algorithm as search strategies to automatically focus the tip of the micropipette. However, both methods require a long moving distance and are prone to tip damage when the tip is driven to the focused plane of adherent cells due to overshooting, which limits the application when the cells in the background have been focused. More specifically, the focusing process of the nanopipette tip can only be done manually to avoid the tip damage when the stage carrying the cells has no *z*-axis freedom and the objective lenses of the microscope are not motorized. Apart from these, the relative vertical positioning between the tip of the micropipette and the cell (proximity detection) is required for automated intracellular sensing because of the highly varied thickness of the adherent cells [29].

Here, we propose an automated intracellular electrochemical sensing system for adherent cells. The number of cell contours (NOCC) obtained from a cell segmentation algorithm was used as the focus algorithm to determine the defocused plane for cell detection. The cell segmentation algorithm integrates the triangle thresholding algorithm and the Otsu thresholding algorithm, which guarantees the unimodality features of the NOCC-based focus curve. The automated cell/nucleus detection is achieved by automatic *z*-axis positioning of adherent cells to reach a defocused plane with the maximal NOCC. Besides, the normalized correlation coefficients during template matching at different *z*-axis positions were utilized as the focus algorithm to autofocus the nanopipette tip without overshooting and tip damage. The nanopipette tip detection was achieved by defining a region of interest containing a defocused nanopipette tip to avoid the time-consuming calculation during template matching and the false matches for cells or impurities. A relative height between the nanopipette tip and the cell surface was detected for the cells in the defocused plane based on ion current feedback, which can avoid the deformation of the soft cells. A nanopipette sensor was fabricated and employed for the specific detection of intracellular ROS. The experimental results demonstrate the high consistency and efficiency of the automatic intracellular electrochemical sensing by the developed system.

## 2. Materials and Methods

### 2.1. Fabrication of Nanopipettes as Sensing Probes

**Figure 1:**
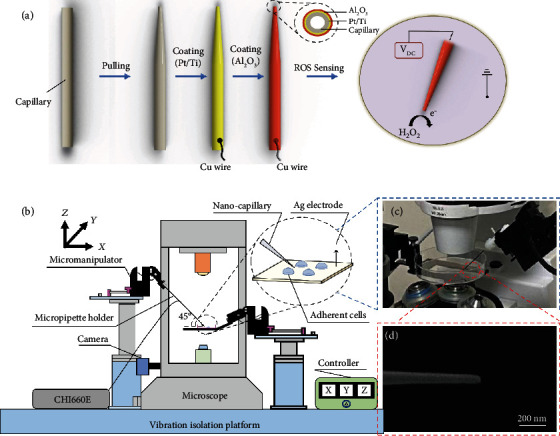
The automated system for intracellular electrochemical sensing. (a) Schematic showing the preparation of nanopipette-based electrochemical nanosensor. (b) Schematic of the automated robotic platform for intracellular electrochemical sensing. (c) A photograph showing the automated positioning of the nanopipette relative to the cell surfaces in the culture dish. (d) Morphological characterization of the nanopipette by electron beam microscope.

Due to the high spatiotemporal resolution and low invasiveness, nanopipette has been a highly versatile platform for precise microinjection [28, 30], nanobiopsies [35, 36], scanning electrochemical microscopy [37], and monitoring chemical/biochemical changes within a single cell [20]. The fabrication of nanopipettes utilizes a laser pipette puller (Glass puller, P-2000, Sutter Instruments) to convert glass capillaries into nanopipettes by following sequential heating and pulling process, as shown in Figure 1(a). Nanopipettes of varying diameters with tip diameters as small as 100 nm can be obtained. Nanopipette-based nanosensor was achieved by sequential electron beam evaporation of titanium (Ti) layer and platinum (Pt) layer, where the Ti layer enhances the adhesion between the glass nanopipette and Pt layer. Atomic layer deposition (ALD) was used to deposit an insulation layer of Al_2_O_3_ (100 nm) for high signal-to-noise ratio intracellular measurements. Finally, focused ion beam (FIB) milling was performed to achieve a consistent tip size and expose the Pt layer for ROS detection. Reactive oxygen species, consisting of radical and nonradical oxygen species formed by the partial reduction of oxygen, have been proved to regulate tumor metastasis and cellular signaling [38, 39]. In this paper, the automated intracellular detection of ROS was achieved. The Pt coating on the nanopipette can catalyze the oxidation reaction of ROS in the cell, where the oxidation current was detected by an electrochemical workstation (CHI660E, CH Instruments).

### 2.2. System Setup

The proposed automated robotic platform for intracellular electrochemical sensing was composed of the electrochemical sensing system, visual feedback system, and motion control system, as shown in Figure 1(b). The electrochemical sensing system, which is mainly responsible for subcellular sensing, consists of the electrochemical workstation and the fabricated nanopipette mounted on a high-precision micromanipulator. An inverted fluorescence microscope (Axio Observer 5, ZEISS) with a CMOS camera (acA4096-40uc, Basler AG, Germany) was used for visual feedback. All images were taken at a 20 × magnification, and each of the captured images was resampled from 4096 pixels × 2168 pixels to 1024 pixels × 542 pixels for faster image processing. The motion control system incorporates two independent four degrees-of-freedom (DOF) micromanipulators (*μ*Mp-4, Sensapex, Finland) with a travel range of 20 mm, a motion resolution of 5 nm, and a maximum speed of 5 mm/s along each degree. Figure 1(c) shows the automated positioning of the nanopipette relative to the cell surfaces in the culture dish. The nanopipette was mounted on one of the micromanipulators. Figure 1(d) shows the microstructure of the nanopipette under an electron beam microscope. The other micromanipulator was used for holding the cell dish. A host computer (CPU: Inter® Core™ i7-8550U) was used to automate the motion control with visual feedback.

The automated detection of adherent cells was evaluated on human umbilical cord vein endothelial cells (HUVEC) and NIH/3T3 cells. In a typical operation, the cell culture dish and nanopipette were mounted on the left and right micromanipulators, respectively. The adherent cells were moved along the *z*-axis to reach the best defocused image plane for cell detection by implementing the NOCC-based focus algorithm. Then, the target sites for cell penetration were identified on the geometrical center of each detected cell contour, and the overall penetration path was optimized. Then, the nonovershoot nanopipette tip positioning was performed to focus and detect the nanopipette tip. Later, the accurate relative vertical position of the nanopipette and the cell surface was obtained based on the ion current feedback. Finally, the nanopipette was controlled to penetrate the cells, and intracellular ROS detection was achieved by following the shortest path.

### 2.3. Cell Detection and Positioning

**Figure 2:**
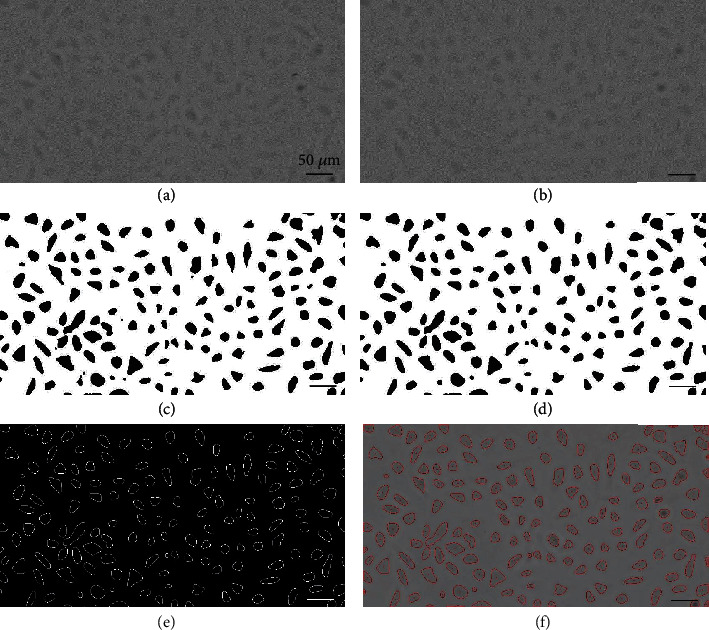
The image processing for cell detection. (a) Original image. (b) Image after Gaussian blurring operation. (c) Image binarization. (d) Image denoise by morphological close operation. (e) Contours of cells. (f) Identification of cell penetration sites overlaid on the original image.

Adherent cells are mostly transparent and exhibit fewer features in the focus plane, which makes it difficult to segment the cells. According to Zernike's phase-contrast method [40], the image of a transparent object can be more explicit for cell detection when slightly defocused under the optical microscope [41]. The contrast of the defocused image of transparent and adherent cells ($C\left(\overset{⟶}{\rho }\right)$) can be written as

(1) $$\begin{array}{c} C\left(\overset{⟶}{\rho }\right)≅\frac{I\left(\overset{⟶}{\rho }\right)-I_{0}}{I_{0}}=\Delta f\Delta n\nabla^{2}h\left(\overset{⟶}{\rho }\right), \end{array}$$

where $\overset{⟶}{\rho }$ and $I\left(\overset{⟶}{\rho }\right)$ represent pixel coordinates and the pixel intensity corresponding to the coordinates, respectively. *I*_0_ is the image intensity of the background. Δ*f* is the defocused distance, which is the distance between the imaging plane and the ideal focus plane along the *z*-axis. Δ*n* represents the refractive index difference between the cell and the medium. $h\left(\overset{⟶}{\rho }\right)$ is the thickness profile of the cell at the corresponding coordinates. The image contrast is proportional to the defocusing distance Δ*f*. Thus, slightly defocusing the image is essential to bring the transparent adherent cells into view. However, the defocused plane selected based on experience cannot maximize the difference between foreground and background, which leads to a poor detection rate of cells. Here, we design a NOCC-based focus algorithm to find the optimal defocused distance by maximizing the detection rate of nonrepeatedly labeled cells.  Algorithm 1 illustrates the procedure of the NOCC-based focus algorithm, and Figure 2 shows the intermediate process.

To avoid the detection of one cell as multiple discrete labeled regions, the original image *I* (Figure 2(a)) was blurred to the image *I*_*G*_ by a Gaussian function *G*(*x*, *y*). Then, the image *I*_*G*_ was transformed into grayscale image *I*_gray_ (Figure 2(b)). Next, an image segmentation algorithm incorporating the triangle thresholding algorithm and the Otsu thresholding algorithm was utilized. The number of cell contours based on the blob analysis after segmentation was used as the focus algorithm. The segmentation algorithm keeps NOCC zero when the cell defocuses negatively and extracts cell regions when the cell defocuses positively. In the triangle thresholding method, a line is constructed between the gray histogram peak and the farthest end of the histogram. In our study, the binarization threshold *T*_otsu_ obtained by the Otsu thresholding algorithm was compared with the gray level *T*_peak_ of the gray histogram peak to determine the constructed line for the triangle thresholding algorithm. When the threshold *T*_otsu_ is greater than *T*_peak_, the constructed line is connected between the peak of the gray histogram and the brighter end of the histogram. The binary image *I*_bin_ can be obtained by the triangle thresholding method, and the connected background region with zero number of cell contours was obtained. When the threshold *T*_otsu_ is smaller than *T*_peak_, the binarization *I*_bin_ (Figure 2(c)) was obtained by the Otsu thresholding algorithm. The developed image segmentation algorithm can shield the effect of the increase of the grayscale difference during negative defocusing and results in a high cell detection rate. Then, the binarized image was denoised by morphological close operation (*I*_close_ (Figure 2(d))). *I*_close_ was then inverted as *I*_inv_. The cell contours *C*_cells_ (Figure 2(e)) were extracted by the OpenCV function findContours(), and the number of cell contours NOCC was determined. Finally, the penetration sites *M*_cells_ were identified by centroid extraction as shown in Figure 2(f).

### 2.4. Detection and Positioning of Nanopipette Tip

**Figure 3:**
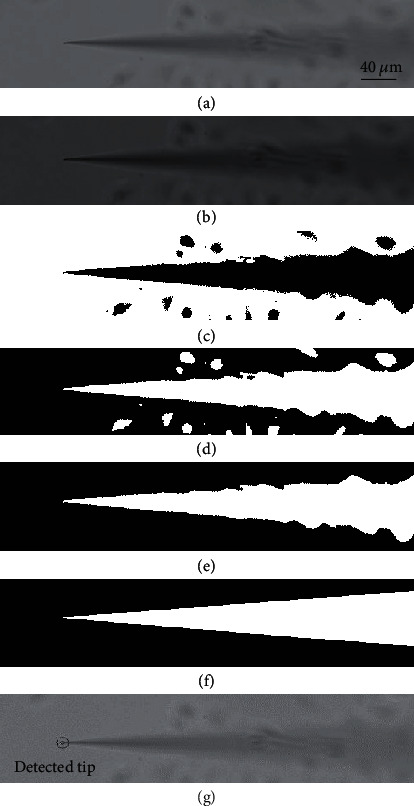
Automated tip detection and positioning. (a) Original image. The scale bar is suitable for the rest of the images. (b) Image after gamma transformation. (c) Image binarization. (d) Image obtained after denoising and inversion. (e) Contour extraction. (f) Contour fitting. (g) Tip detection.

In a typical procedure, the region of interest (ROI) of the nanopipette tip during the autofocusing was extracted to reduce the time consumption during template matching by thresholding and finding contours [42]. The detection of the nanopipette tip needs to avoid failure detection where the impurities or cells in the background are more notable than the nanopipette tip when the nanopipette tip is far away from the focus plane. As shown in Figure 3, the initial image (Figure 3(a)) was enhanced by gamma transformation to make the features of the nanopipette tip more obvious (Figure 3(b)). Then, the image was binarized by the Otsu thresholding method and denoised by the morphological close operation (Figure 3(c)). Since the tip area is darker than the background, the image was inverted (Figure 3(d)), and the longest contour of the nanopipette tip was obtained (Figure 3(e)). The contour of the nanopipette tip was fitted by the OpenCV function approxPolyDP(), which conforms to a triangular contour (Figure 3(f)). The presence of a triangular feature is used as a reference for the successful detection of the nanopipette tip. If the fitted contour is triangle-shaped, the vertex of the triangle will be used as the tip of the nanopipette. To further analyze the weak feature of the nanopipette quantitatively, the relationship between defocus distance and the successful detection of the nanopipette tip was studied. It was found that the tip detection algorithm could successfully detect the nanopipette tip when the defocus distance is smaller than approximately 110 *μ*m. If the fitted contour is not triangle-shaped, the tip moves continually towards the focus plane. Finally, the tip of the nanopipette can be obtained (Figure 3(g)).

The depth from focus (DFF) method and the depth from defocus (DFD) method are two typical passive autofocusing methods that are currently studied. The DFD method is not suitable for this study because the initial defocused depth of the nanopipette is larger than the working range of the DFD method. The feature of the nanopipette is too weak to be recognized. Besides, the DFF method lacks a prediction mechanism of the tip-sample distance to avoid any collision damage to the tip of the nanopipette. Thus, we propose a nonovershoot autofocusing method based on template matching to realize the three-dimensional tip positioning of the nanopipette.

The template matching using normalized correlation coefficient was used to achieve nonovershoot tip autofocusing. When the tip of the nanopipette was moved downwards to the focus plane of adherent cells, the image *I* was compared with the precollected template image of the focused nanopipette tip *T*. The similarity *S*(*x*, *y*) of these two images was calculated by

(2) $$\begin{array}{c} S\left(x,y\right)=\frac{\sum_{x^{\prime },y^{\prime }}\left(T^{\prime }\left(x^{\prime },y^{\prime }\right)\cdot I^{\prime }\left(x+x^{\prime },y+y^{\prime }\right)\right)}{\sqrt{\sum_{x^{\prime },y^{\prime }}T^{\prime }{\left(x^{\prime },y^{\prime }\right)}^{2}\cdot \sum_{x^{\prime },y^{\prime }}I^{\prime }{\left(x+x^{\prime },y+y^{\prime }\right)}^{2}}}, \end{array}$$

where

(3) $$\begin{array}{c} T^{\prime }\left(x^{\prime },y^{\prime }\right)=T\left(x^{\prime },y^{\prime }\right)-\frac{1}{w\cdot h}\cdot \underset{x^{\prime \prime },y^{\prime \prime }}{\sum }T\left(x^{\prime \prime },y^{\prime \prime }\right),\\ I^{\prime }\left(x+x^{\prime },y+y^{\prime }\right)=I\left(x+x^{\prime },y+y^{\prime }\right)-\frac{1}{w\cdot h}\cdot \underset{x^{\prime \prime },y^{\prime \prime }}{\sum }I\left(x+x^{\prime \prime },y+y^{\prime \prime }\right). \end{array}$$

In the above equations, *w* and *h* are the width and height of the template image, respectively. The tip of the nanopipette can accurately stop above the focus plane when reaching an appropriate threshold of the similarity.

### 2.5. Proximity Detection between the Nanopipette and the Cell Surface

**Figure 4:**
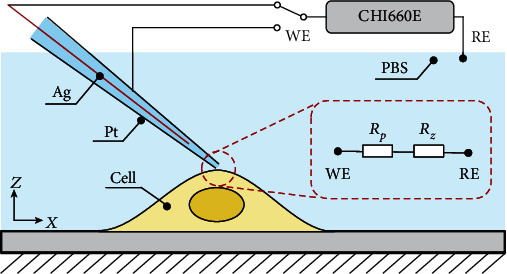
Proximity detection based on ion current feedback.

The nonovershoot autofocusing of the nanopipette tip cannot accurately determine the relative vertical height between the nanopipette tip and the top surface of the cell. Here, a proximity detection based on ion current feedback was used to determine the relative vertical positions between the nanopipette tip and the cell surface. The schematic for proximity detection is shown in Figure 4. An Ag/AgCl reference electrode (RE) was immersed in the phosphate buffer solution (PBS). The Ag/AgCl working electrode (WE) was inserted into the nanopipette backfilled with PBS solution. When the nanopipette approaches the cell surface, the ion current (*I*) that flows between the WE and RE begins to decrease because the nanopipette tip is gradually occluded. The relationship between nanopipette tip-cell distance (*d*) and ion current can be described as

(4) $$\begin{array}{c} I\left(d\right)=\frac{U}{R_{p}+R_{z}}\approx I_{\infty }{\left(1+\frac{\left(3/2\right)\ln\left(r_{o}/r_{i}\right)r_{i}r_{e}}{hd}\right)}^{-1}, \end{array}$$

where *I*(*d*) represents the distance-dependent ion current and *U* is the applied potential between the WE and RE [43]. Besides, the resistance of the nanopipette *R*_*p*_ (depends on the geometry of the pipette and the conductivity of the electrolyte solution) and the access resistance *R*_*z*_ (depends on the conductivity of the electrolyte solution and the gap formed between the tip and interface) form the total resistance between the WE and RE. The steady-state ion current (*I*_∞_) can be measured only depending on (*R*_*p*_) resistance, while the nanopipette tip is away from the sample surface. *r*_*i*_ and *r*_*o*_ are the internal radius and the outer radius of the nanopipette tip, respectively. *r*_*e*_ is the internal radius of the nanopipette base, and *h* is the nanopipette height. As the nanopipette tip moves closer to the cell surface, the access resistance (*R*_*z*_) increases quickly and hence decreases the ion current. The cell-nanopipette tip proximity detection is a noncontact approach, which can avoid the deformation of the soft cells. Besides, the noncontact approaching action to the cell can avoid unintentional mechanotransduction processes, where physical cues may induce changes in intracellular biochemistry and gene expression [44, 45]. Besides, a maximum protection or contamination avoidance of the nanopipette tip can be ensured during the whole automated electrochemical sensing process by the robotic nanopipettes. In the empirical practice of the automated nanopipette probing, such as scanning ion conductance microscopy, the position of *z*-dimension actuator is recorded as the height of the sample at the point where the current reduction of 0.25-1% is identified [46–48]. To further avoid the false identification by current fluctuation due to measurement error and environmental disturbances, a 2% current reduction was set as the threshold to guarantee high resolution and stable proximity detection, which is still a noncontact approaching process between the nanopipette tip and sample [49].

## 3. Results and Discussions

### 3.1. *z*-Axis Positioning of Adherent Cells

**Figure 5:**
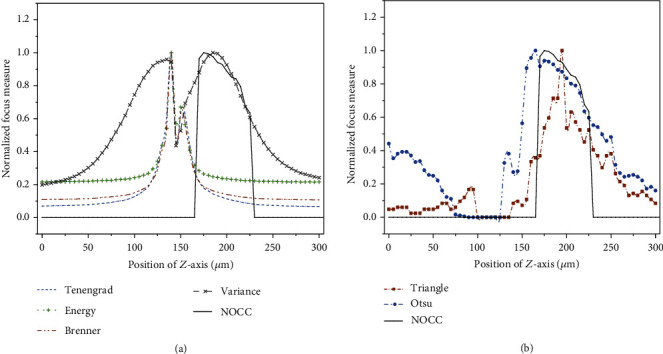
Comparison of different focus algorithms. (a) Comparison between the traditional focus algorithms and the NOCC-based focus algorithm. (b) Comparison of the NOCC-based focus algorithm with the triangle thresholding method or the Otsu thresholding method.

Automatic *z*-axis positioning of the adherent cells is of great importance for cell detection with a high positive rate. To evaluate the performance of automatic *z*-axis positioning, the NOCC-based focus algorithm was experimentally compared with the other four kinds of traditional focus algorithms (Tenengrad [50], Energy [51], Brenner [52], and Variance [50]). The different focus curves were normalized, as shown in Figure 5. Several quantitative metrics (accuracy, range, the number of false maxima, and noise level) were used to evaluate the performance.

The focus plane of adherent cells determined by traditional focus algorithms renders cell detection difficult. Here, the successful rate of cell detection was used to evaluate the accuracy of the proposed focus algorithm, where the best defocused plane corresponds to the maximal NOCC. The traditional range measures the distance between two local minima containing a global maximum. It is easier to search for an accurate maximum with a large distance range. However, the focus plane of adherent cells corresponds to the local minimum of the traditional focus algorithm. Thus, the distance between the two neighboring maxima of the focus plane was used as the range to evaluate the performance of the traditional focus algorithms. The number of false maxima indicates the number of local maxima on the focus curve. The noise level is represented by the sum of the squares of the second derivatives of the focus curve. In this study, the ideal range and number of false maxima are 300 and 0, respectively. The quantitative comparison of different focus algorithms is shown in Table 1.

In the experiment, five sets of 60 images were collected with a step size of 5 *μ*m to evaluate the focus algorithms and cell detection for HUVEC and NIH/3T3 cells. The evaluation results are shown in Figure 5(a). The traditional focus algorithms lack unimodal characteristics and form local minima during cell focusing, which makes the *z*-axis positioning challenging for adherent cells. In contrast, the maximum of the NOCC-based focus curve corresponds to a defocus plane that enhances the gray difference between the cell and the background, which can better detect the distinct cell regions and guarantee a high detection rate for cell/nucleus. As seen in Figure 5(a), the maximum of the Variance algorithm is close to that of the NOCC-based focus algorithm. However, only the NOCC-based focus algorithm can avoid the local maximum in the range of 0-165 *μ*m and presents unimodal characteristics. The feature indicates an excellent antinoise ability of the NOCC-based focusing method.

Besides, unimodal focus curves cannot be obtained by the triangle thresholding method or the Otsu thresholding method, as shown in Figure 5(b). In the range of 165-230 *μ*m, the characteristics of the NOCC-based focus curve were consistent with those based on the Otsu method because the same algorithm was used. The inconsistency in Figure 5(b) is caused by the normalization operation of the focus curve. However, the triangle thresholding and Otsu threshold methods cannot guarantee the unimodal characteristics of the focus curve because the negative defocusing can also increase the grayscale difference between the foreground (cells) and background in the range of 0-100 *μ*m. In the experiment, the maximal number of detected cells based on the triangle thresholding method (84) is significantly lower than that obtained by the NOCC-based focus algorithm (170).

As shown in Table 1, the range of the traditional focus algorithms is less than the ideal range (300 *μ*m). The result indicates that the positioning range satisfying the unimodal property is small, which causes the failure in autofocusing of the adherent cells. The range of the NOCC-based focus algorithm is significantly larger than that of other focus algorithms. Meanwhile, the number of false maxima of the NOCC-based method is far less than that of other focus algorithms. The range of the NOCC-based method is still less than the ideal value because of noise. However, the noise level of the NOCC-based focus algorithm (0.006) is less than the allowable noise level in the dynamic curve fitting search strategy (0.648) with a corresponding variance of 1 [26], which demonstrates that the global maximum of the NOCC-based focus algorithm can be searched accurately.

### 3.2. Cell Detection

**Figure 6:**
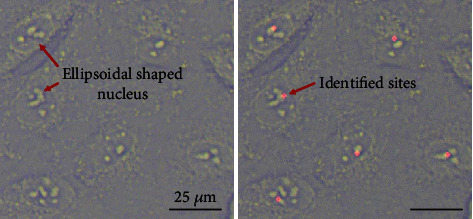
The ellipsoidal-shaped nucleus as the ground truth for cell detection.

The efficiency of cell detection was evaluated with HUVEC and NIH/3T3 cells under the defocused plane. The ellipsoidal-shaped nucleus from a focused image was used as the ground truth, as shown in Figure 6. The cell detection was evaluated by two quantitative metrics: hit ratio and error rate. The hit ratio defines the number of correctly detected cells out of the total number of cells. The error rate equals the percentage of the number of off-target points and multiple labeled cells over the total cell number. The quantitative results were presented in terms of true-positive rate (TPR) and false-positive rate (FPR), which are expressed as

(5) $$\begin{array}{c} \text{TPR}=\frac{\text{TP}}{\text{TP}+\text{FN}},\\ \text{FPR}=\frac{\text{FP}}{\text{TP}+\text{FN}}, \end{array}$$

where TP represents the number of accurately identified cells and FN indicates the number of undetected visible cells. The sum of TP and FN represents the total cells in the field of vision. FP refers to the falsely detected cells. In an ideal case, the TPR and FPR are 100% and 0%, respectively, where all visible cells are detected.

The experiments were performed based on an evaluation set made of 10 images containing a total of 755 HUVEC cells and 593 NIH/3T3 cells. Cells with invisible nucleus features at the far end of the image were not counted. The TPR of HUVEC and NIH/3T3 cells was 97.44% and 93.47%, respectively. The lower TPR for NIH/3T3 cells was caused by their irregular shapes and overlapping. Compared with the cell detection rate reported by Pan [30] and Becattini [32], the TPR by the proposed method, as shown in Table 2, is higher, which shows better cell/nucleus identification. Hence, the defocused plane determined by the NOCC-based focus algorithm is more reliable for cell detection. The lower FPR indicates a lower probability of incorrect cell labeling and cell penetration. In addition, the average cell number in the field of vision was 129.8 in this study, which is 4.2 times of the cell number in Pan's study, which significantly improves the efficiency of intracellular electrochemical sensing. The cell positioning algorithm is also suitable for intracellular electrochemical sensing of multiple cells. However, the cell positioning algorithm, by determining the cell contours, can only localize the nucleus, thus hindering intracellular electrochemical analysis to the other regions inside the living cell, such as the cytoplasm.

### 3.3. Nonovershoot Tip Positioning

**Figure 7:**
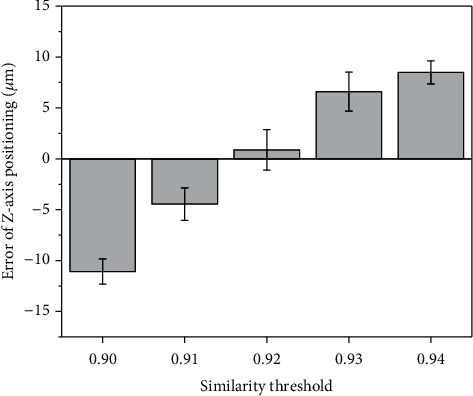
The nonovershoot positioning error with different similarity threshold.

The nonovershoot tip positioning algorithm was evaluated by giving five different similarity thresholds under the background of the defocused cells. The tip positioning error was obtained by referring to the coordinates given by the micromanipulators, and the 95% confidence interval was used as the error bar, as shown in Figure 7. When the similarity threshold is 0.9, the *z*-axis positioning error is −11.07 ± 1.24 *μ*m, and the tip stops at 11.07 *μ*m before the focus plane. When the threshold is 0.92, the *z*-axis positioning error is 0.88 ± 2.00 *μ*m, and the tip may be stopped on either side of the focus plane. The tip will pass over the focus plane with a *z*-axis positioning error of 8.50 ± 1.13 *μ*m when the threshold is 0.94. The tip positioning goes from nonovershooting to overshooting with the increase of similarity threshold. One may raise the question of why the similarity of the template matching during overshooting is higher than that of the focused plane. That is because the template image of the nanopipette tip composed of a black tip area and white background can lead to a higher similarity when the nanopipette tip is over the focus plane slightly. Compared with the tip autofocusing of nanopipettes by detecting the body firstly, the proposed nonovershoot tip positioning method can avoid tip damage.

In addition, the robustness of the nonovershoot tip positioning algorithm was separately evaluated on the Pt-coated nanopipette for electrochemical sensing (E-capillary) and the pristine glass nanopipette for microinjection (M-capillary) under the focused cell background (F-cells) and the defocused cell background (D-cells). The experimental errors were represented by 95% confidence intervals (CI), as shown in Table 3. The *z*-axis positioning error of M-capillary is -1.07 *μ*m and -1.95 *μ*m, where the similarity threshold was set to 0.85 (less than 0.92) because of the low contrast of the transparent M-capillary tip. For the condition of E-capillary, the threshold is 0.91. The defocused cell background avoids the interference of impurities in the focusing image, advancing the stop position of the tip by 3.07 *μ*m. In conclusion, the nonovershoot tip positioning algorithm can be used for the opaque and pristine nanopipettes under the focused and defocused cell background.

### 3.4. Intracellular Electrochemical Sensing

**Figure 8:**
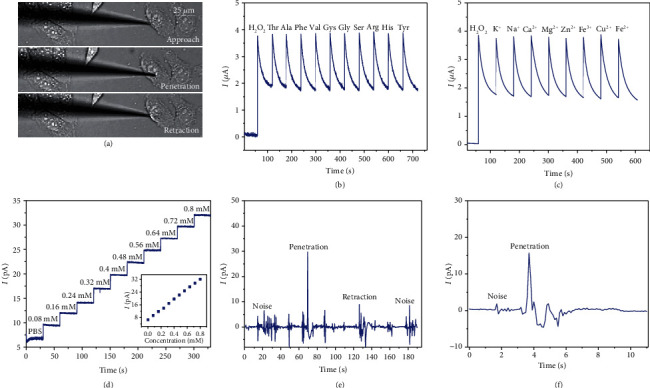
The intracellular ROS detection. (a) Optical images of the nanopipette approaching to the cell, cell penetration, and nanopipette retraction. (b) Current recordings of ROS when different kinds of amino acids are added to the solution. (c) Current recordings of ROS when the Pt electrode is immersed in the solution of different ions. (d) Current response with different concentrations of hydrogen peroxide. (e) The current recording of ROS for zebrafish embryos with band-pass filtering. (f) The ROS current detection from MCF-7 cell with band-pass filtering.

To quantitatively understand the function of biological systems, the electrochemical monitoring of intracellular ROS requires a nanopipette-based sensor that is selective and sensitive to targeted analytes. Thus, the selectivity of the Pt for intracellular electrochemical sensing of hydrogen peroxide (H_2_O_2_) was evaluated by using a Pt working electrode with a 1 mm diameter and an Ag/AgCl reference electrode. The working electrode and reference electrode were placed in the PBS solution, and the current was recorded. As shown in Figure 8(b), the current increased rapidly and then decreased slowly to 1.8 *μ*A when H_2_O_2_ was added to the solution. In addition, a sudden overshoot of current was observed when adding different amino acids such as Thr, Ala, Phe, Val, Cys, Gly, Ser, Arg, His, and Tyr. However, the current was finally stabilized to 1.8 *μ*A, demonstrating that the Pt microelectrode is capable of selective quantification of ROS. Moreover, the current recorded against different ionic disruptors further proves the selectivity of the Pt (Figure 8(c)).

Finally, the automated intracellular electrochemical sensing of ROS was performed by automated cell detection, tip positioning, cell penetration, and nanopipette retraction from the cell. Two types of cells, namely, zebrafish embryos and human breast cancer cells (MCF-7), were selected for the study. Due to the small volume of adherent cells (MCF-7), 30 minutes of cellular inflammation stimulation was implemented by adding 1.0 mg/mL phorbol myristate acetate (PMA) to increase ROS before detecting the intracellular ROS level. Figure 8(d) shows that the relationship between the concentration of H_2_O_2_ and current was quantified by adding different concentrations of H_2_O_2_ in PBS solution. The currents increased with the concentration of H_2_O_2_, and current amplitude was proportional to the concentration of hydrogen peroxide within the range from 0.08 mM to 0.8 mM. In addition, the burst signal during penetration with a band-pass filter demonstrates intracellular ROS sensing for zebrafish embryo and MCF-7 cell, respectively, as shown in Figures 8(e) and 8(f). The variation of ROS signals during the penetration of both types of cells can be observed clearly, which indicates that the fabricated nanopipette sensor is capable of intracellular ROS detection and demonstrates the remarkable capabilities of automated intracellular electrochemical sensing. As the next step, the automated intracellular electrochemical sensing will leap forward in the advancement of multiple intracellular signal detections and manipulation of cell organelles based on real-time measurements of intracellular activities. Besides, automated intracellular electrochemical sensing may provide a reliable approach for the detection of a low concentration of biomarkers and the dynamic analysis of intracellular content in living single cells.

## 4. Conclusion

In this study, our robotic intracellular electrochemical sensing for adherent cells presents a simple, inexpensive, and nondestructive approach for living single cells with a high spatial resolution at a subcellular level. The developed system can accurately achieve cell detection, three-dimensional nonovershoot positioning of the nanopipette tip, cell approaching and proximity detection between nanopipette tip and cell surface, and cell penetration and detection of the intracellular reactive oxygen species (ROS). A focus algorithm based on the number of cell contours is proposed for automatic *z*-axis positioning of adherent cells, in which a cell segmentation algorithm integrating the triangle thresholding algorithm and the Otsu thresholding algorithm is used to guarantee the unimodality features of the focus curve. The automated cell/nucleus detection was achieved in a defocused plane with the maximal number of cell contours. The cell detection was performed on HUVEC and NIH/3T3 cells, which demonstrated an average of 95.65% cell targeting rate and the unimodal features of the proposed focus algorithm. Compared with the defocused plane determined by experience [32], our results prove that the defocused plane determined based on the NOCC-based focus algorithm can significantly improve the cell detection rate, which is significant to improve the efficiency of high-throughput cell sensing. The three-dimensional nonovershoot tip positioning of the nanopipette was realized based on template matching, and the experimental results demonstrated that the nonovershoot tip positioning of the Pt-coated and pristine glass nanopipettes can be achieved under the focused and defocused cell background. Besides, ion current signal was used to detect the relative vertical positions between the nanopipette tip and the cell without cell deformation. The cell penetration and electrochemical detection of ROS were evaluated by human breast cancer cells and zebrafish embryo cells, and the variation of ROS signals indicates the capability of intracellular ROS detection. The nanopipette-based electrochemical sensing system makes it possible to detect the intracellular ROS in tens to hundreds of cells, demonstrating the potential for high-throughput intracellular sensing. Besides, the automated nanopipette-based electrochemical sensing system can achieve spatial manipulation of cells and organelles with self-sensing feedback when coupled with other nanopipette-based micromanipulation techniques, such as dielectrophoretic micromanipulation for selection, isolation, and positioning of cells and organelles. The proposed system will also have important applications in lineage tracing for developmental biology and high-resolution localization of organelles in living single cells for investigating the specific causes of diseases and the development of novel therapeutics.

## Figures and Tables

**Algorithm 1:**
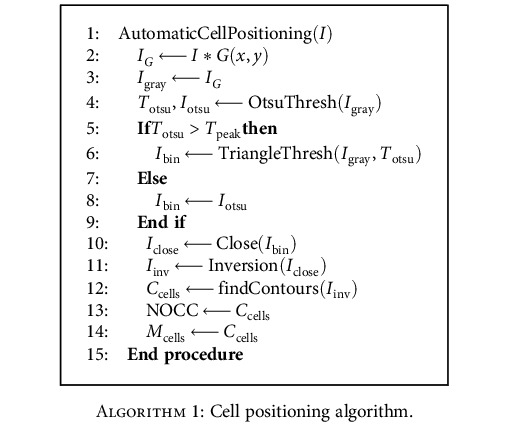
Cell positioning algorithm.

**Table 1:** Quantitative comparison of different focus algorithms.

Metrics	Ideal	NOCC-based method	Energy	Tenengrad	Brenner	Variance	Triangle	Otsu
Range (*μ*m)	300	247	12.5	13.5	13	33	60.5	42.5
False max	0	0.6	8	2.7	4.6	1.6	6.2	8.4

**Table 2:** Comparison of cell detection by different methods.

Methods	Cell line	TP	FN	FP	TPR	FPR
Proposed method	HUVEC	145	3.8	11	97.44%	7.39%
Proposed method	NIH-3T3	114.6	8	9.6	93.47%	7.83%
Average	HUVEC, NIH-3T3	129.3	5.9	10.3	95.65%	7.59%
Pan [30]	MC3T3-E1	30.6	8.9	3.4	89.95%	26.20%
Becattini [32]	CHO-K1, HEK	\	\	\	78.6%	18%

**Table 3:** Results of the nonovershoot tip positioning.

Condition	*z*-axis error (*μ*m)
M-capillary and F-cells	–1.07 ± 1.18
M-capillary and D-cells	–1.95 ± 1.55
E-capillary and F-cells	–1.37 ± 0.97
E-capillary and D-cells	–4.44 ± 1.59

## Data Availability

Research data used to support the findings of this study are available from the corresponding author upon request.
